# ^18^F-fluorodeoxyglucose positron emission tomography-computed tomography as a screening tool for second primary cancers in cancer patients

**DOI:** 10.18632/oncotarget.21444

**Published:** 2017-09-30

**Authors:** Yang Liu, Jie Ma, Yingxin Liu

**Affiliations:** ^1^ Department of Radiation Oncology, Cancer Hospital of Guangxi Medical University, Nanning, People's Republic of China; ^2^ Department of Radiology, Cancer Hospital of Guangxi Medical University, Nanning, People's Republic of China

**Keywords:** second primary cancers, PET-CT, staging

## Abstract

**Background:**

We performed a meta-analysis to evaluate the value of ^18^F-fluorodeoxyglucose positron emission tomography-computed tomography (^18^FDG PET-CT) for the detection of second primary cancers in cancer patients.

**Results:**

This present study analyzed a total of 6 selected studies (1374 patients). The sensitivity and specificity of PET-CT were 0.84 (95% confidence interval [CI] = 0.66 to 0.93), and 0.98 (95% CI = 0.97 to 0.98). Area under the curve was 0.98 (95% CI = 0.96 to 0.99).

**Methods:**

Studies were systematically searched for relevant PET-CT original articles in the MEDLINE and EMBASE databases. We calculated the pooled sensitivity, specificity, and likelihood ratios for ^18^FDG PET-CT. We also constructed the summary receiver-operating characteristic curve for ^18^FDG PET-CT.

**Conclusions:**

^18^FDG PET-CT has high sensitivity and specificity for the detection of second primary cancers in cancer patients.

## INTRODUCTION

The emergence of second primary cancers (SPCs) is one of the important prognostic factors in cancer patients. SPCs are the leading cause of treatment failure and death in cancer patients with early-stage disease. The early detection of SPCs is essential to reduce the mortality associated with SPCs, particularly in patients without the symptoms that are indicative of a SPC.

Conventional imaging modalities such as simple radiography and computed tomography are not suitable for the detection of SPCs because of the relatively low sensitivity and limited field of coverage [1–4]. Integrated positron emission tomography-computed tomography (PET-CT) could provide more anatomical details for the PET images, which may provide new insights for screening SPCs. Although some studies have reported the use of ^18^FDG PET-CT for the detection of SPCs, the accuracy of PET-CT remains to be controversial [1–6]. Here, we performed a meta-analysis to evaluate the accuracy of ^18^FDG PET-CT for the detection of SPCs in cancer patients at staging.

## RESULTS

### Literature identification

**Figure 1:**
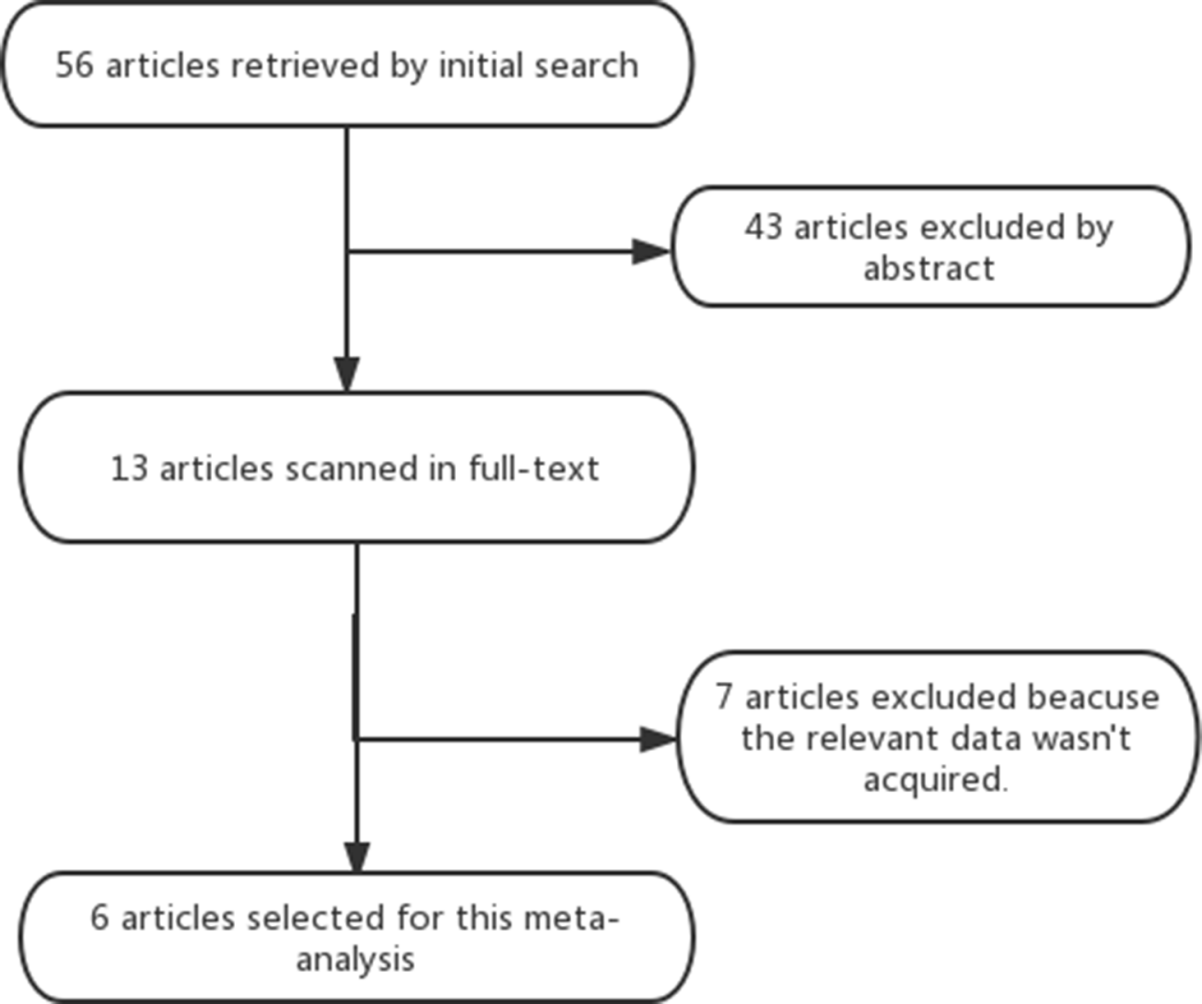
Shows the flow chart of the search for eligible studies

The electronic search yielded 56 articles; 43 articles were excluded by reading the abstract because they did not present any diagnostic information. We screened in 13 full-text articles and rejected 7 full-text articles. 6 articles [1–6] were eligible for meta-analysis (Figure 1). Four (66.7%) of the 6 studies stated that they were prospective. The clinical and imaging characteristics of all six included studies were shown in Table 1. A total of six studies (1350 patients) were analyzed for the accuracy of ^18^FDG PET-CT for the detection of SPCs, including 501 head and neck cancer patients with, 472 esophageal cancer patients, 277 lung cancer patients, 13 gastric cancer patients, and 87 patients with other subtypes of cancer. Of all six studies, 93.6% (1263/1350) of the first primary cancers are carcinomas of the upper aerodigestive tract. In five of all six studies [1–5], the results of ^18^FDG PET-CT was stated to have been assessed in a qualitative manner. And there was still one study [6] in which the assessing manner was both quantitative and qualitative.

**Table 1:** The clinical and imaging characteristics of all six included studies

Author	Year	Origin	No. of Patients	Design	Locations of the first cancer	Age(y)	PET-CT Technique			Follow-up time (month)	Locations of secondary primary cancer
							Dose	CE-CT	Imaging interpretation		
Choi JW(1)	2005	Korea	547	Prospective	Lung (277), Esophagus (113), Head and Neck (57), Stomach (13), Others (87)	60.5 (mean)	370MBq	No	qualitative	9.2±5.2	Lung (5), Esophagus (2), Head and Neck (10), Stomach (5), Colorectium (3), Gallbladder (1).
Chen SH(2)	2013	Taiwan	359	Retrospective	Esophagus(359)	30-80:348 >80:11	370MBq	No	qualitative	Not Reported	Head and Neck (10), Colorectium (2), Liver (1), Kidney (1)
Hoshikawa H(5)	2013	Japan	88	Prospective	Head and Neck(88)	40-83	3.5MBq/kg	No	qualitative	≥6	Lung (1), Esophagus (1), Colorectium (2), Pancreas (1)
Kim JW(3)	2013	Korea	119	Prospective	Head and Neck(119)	20-83	370-555MBq	No	qualitative	≥18	Lung (1), Esophagus (1), Head and Neck (2), Prostate (1)
Seepage Y(4)	2013	Japan	170	Retrospective	Head and Neck(170)	30-89	222–333MBq	No	qualitative	≥12	Lung (7), Esophagus (1), Head and Neck (1), Stomach (1), Prostate (1) Colorectium (2), Liver (1) Breast (1), lymphoma (1)
Park MJ(6)	2017	Korea	67	Prospective	Head and Neck(67)	49-64	370-555MBq	No	Both qualitative and quantitative	≥18.3	Head and Neck (1), Prostate (1)

### Study quality

The results of quality assessment for all six studies was shown in Table 2. For only one study [1], the item of patient selection (risk of bias and applicability concerns) was assessed as low-risk. For all six studies [1–6], the results of ^18^FDG PET/PET-CT were interpreted without any knowledge of the gold standard. But the gold standard wasn't executed without any knowledge of the results of ^18^FDG PET/PET-CT in all six studies [1–6].

**Table 2:** QUADAS-2 results for all six included studies

Studies	Risk of bias				Applicability concerns		
	Patient selection	Index test	Reference standard	Flow and timing	Patient selection	Index test	Reference standard
Choi JW(1)	_	_	+	_	_	_	_
Chen SH(2)	+	_	+	_	+	_	_
Hoshikawa H(5)	+	_	+	_	+	_	_
Kim JW(3)	+	_	+	_	+	_	_
Seepage Y(4)	+	_	+	_	+	_	_
Park MJ(6)	+	_	+	_	+	_	_

#“+” = High-risk, “_”= Low-risk.

### Diagnostic accuracy of ^18^FDG PET-CT

**Figure 2:**
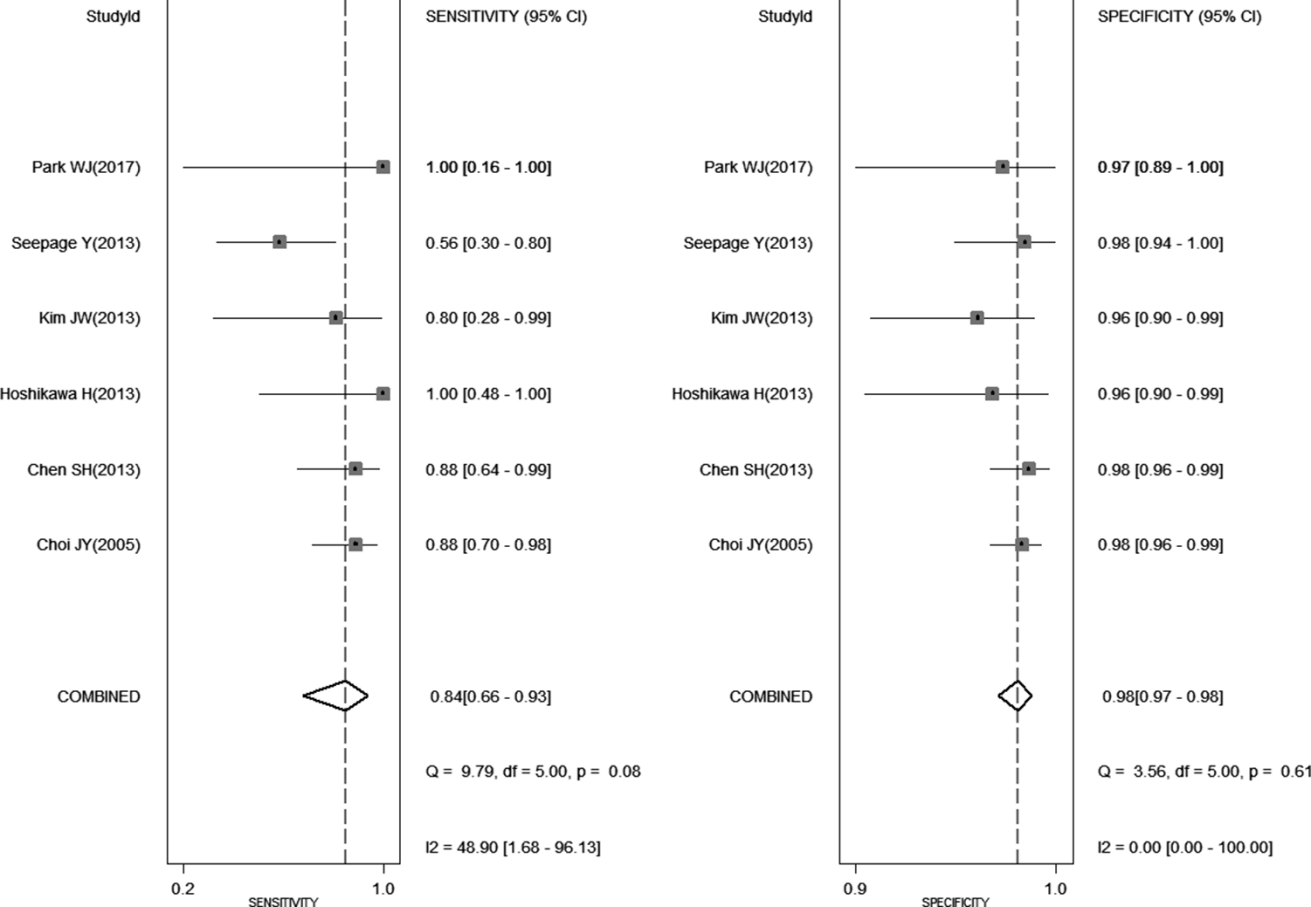
Shows the forest plot of sensitivity and specificity for ^18^FDG PET-CT in the detection of second primary cancers

Figure 2 shows the forest plot of sensitivity and specificity for ^18^FDG PET-CT in the detection of SPCs. When considering all 6 studies (1350 patients) [1–6], the pooled sensitivity, specificity, positive likelihood ratio (PLR), and negative likelihood ratio (NLR) with 95% confidence interval for PET-CT were 0.84 (0.66 - 0.93), 0.98 (0.97 - 0.99), 35.8 (24.2 - 53.2), and 0.16 (0.07 - 0.38), respectively. When considering 4 studies about head and neck cancer (444 patients) [3–6], the pooled sensitivity, specificity, PLR, and NLR with 95% confidence interval for PET-CT were 0.80 (0.41 to 0.96), 0.97 (0.94 to 0.98), 25.3 (13.7 - 46.5), and 0.20 (-0.05 - 0.84), respectively.

**Figure 3:**
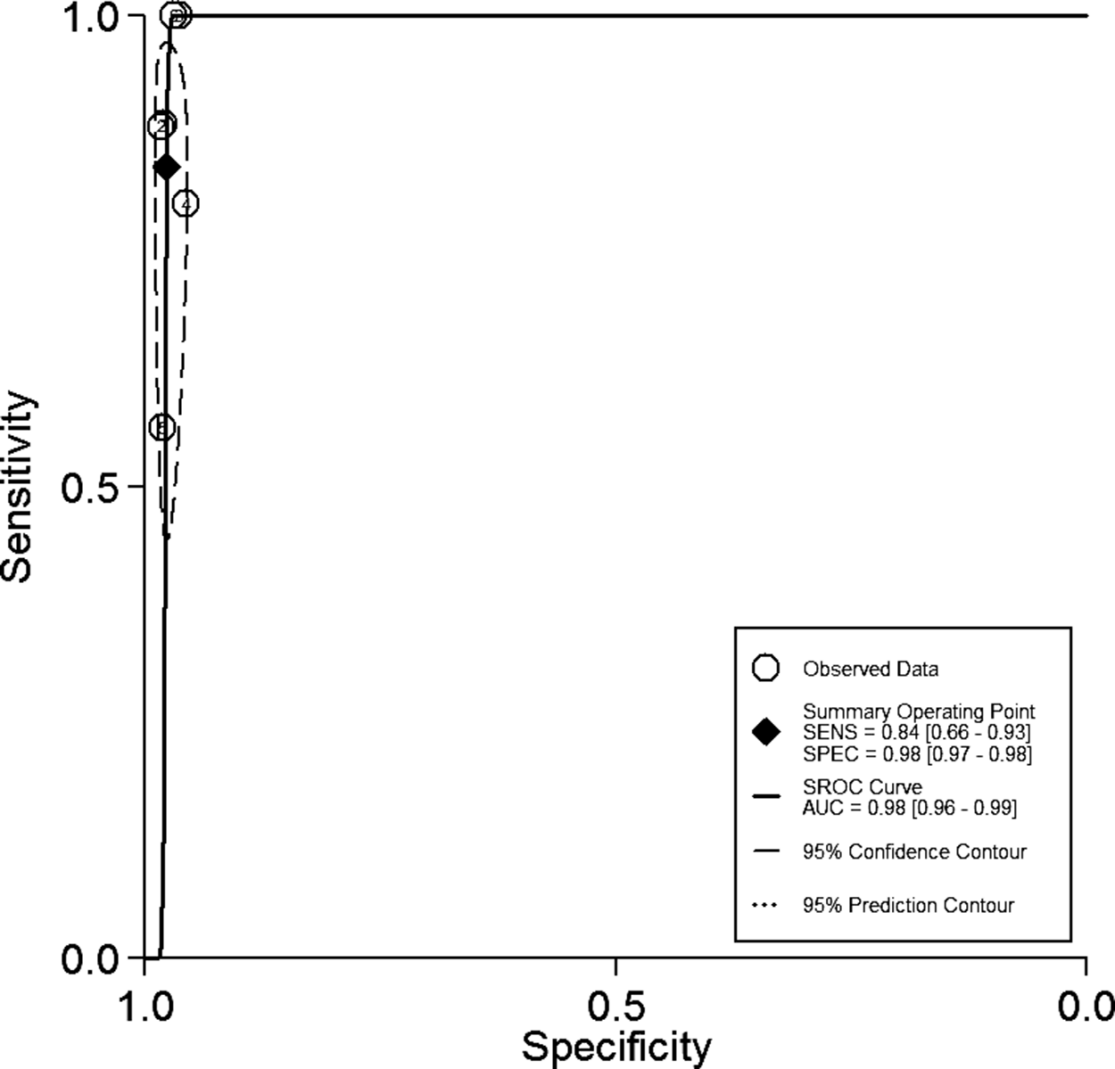
Shows the SROC curve for ^18^FDG PET-CT in the detection of second primary cancers

Figure 3 shows the SROC curve for ^18^FDG PET-CT in the detection of SPCs. The results showed that overall weighted area under the curve with 95% confidence interval was 0.98 (0.96 - 0.99).

When the prevalences of SPCs in cancer patients were assumed to be 5%, 10%, and 15%, the negative predictive values for ^18^FDG PET-CT were 0.99, 0.98, and 0.97, respectively.

## DISCUSSION

The malignancies of the upper aerodigestive tract (oral cancer, pharyngeal cancer, laryngeal cancer, esophageal cancer, gastric cancer, and lung cancer, etc.) have an increased risk of SPCs, which has an incidence of 5% to 10% [1–8]. The most well-known sites for SPCs are also aerodigestive tract organs, such as the oral cavity, pharynx, larynx esophagus, stomach and lung [1–8]. The early detection of SPCs is crucial for choosing the most effective management strategies.

Conventional imaging modalities had limited field of coverage and the relatively low sensitivity for detecting SPCs. Conventional imaging modalities on their own can miss early lesions of SPCs due to normal variation and the mobility of body structures [1]. Integrated PET-CT can provide both the metabolic and anatomic information of a cancer. In the previous study, ^18^FDG PET-CT showed a high sensitivity of 88.5% (23 of 26 patients) in detecting SPCs, which was significantly higher than the 61.5% (16 of 26) from a conventional staging work-up. In this meta-analysis, 6 studies about the clinical use of PET-CT for screening SPCs (1350 patients) were included. And the weighted overall estimates of sensitivity and specificity for PET-CT were 0.84 (95% CI = 0.66 to 0.93) and 0.98 (95% CI = 0.97 to 0.99). This meta-analysis showed that PET-CT can be used as an effective diagnostic tool for detection of second primary cancers. An additional PET-CT is essential for ruling out the presence of SPCs when abnormal findings of conventional imaging modalities are indicative of SPCs. But ^18^FDG PET-CT has a lower sensitivity for screening early-stage carcinomas of digestive tract (esophageal cancer, gastric cancer, intestinal cancer, and colorectal cancer [1–6].

The negative predictive value is a single indicator of diagnostic test about the probability that a patient does not have SPCs when the results of PET-CT are negative. This meta-analysis showed that the negative predictive values for ^18^FDG PET-CT were 0.99, 0.98, and 0.97 when the prevalences of SPCs in cancer patients were assumed to be 5%, 10%, and 15%. When the prevalence of SPCs in cancer patients were assumed to be 5-15%, ^18^FDG PET-CT is very informative lowering the probability of disease to as low as 1-3% when the results of PET-CT are negative.

There were several limitations in this meta-analysis. First, not all included studies had a prospective design. The retrospective studies may have some inevitable limitations. For example, the imaging interpreters may have known some results of conventional imaging before interpreting PET-CT. Second, the publication bias from positive results is a major concern because of the discarded tendency of studies with nonsignificant results. The funnel plot of this meta-analysis was not performed because the number of included studies is only six. Third, there was no single imaging strategy for the follow-up of SPCs in all six studies, which may have affected the accurate evaluation of PET-CT. Fourth, 93.6% of the first primary cancers in this meta-analysis are carcinomas of the upper aerodigestive tract. The accuracy of ^18^FDG PET-CT from this meta-analysis can be applied only to carcinomas of the upper aerodigestive tract.

In conclusion, ^18^FDG PET-CT has high sensitivity and specificity for screening SPCs in cancer patients. Further large and prospective studies are needed to evaluate the value of PET-CT for the detection of SPCs sites in patients with other subtypes of cancer.

## MATERIALS AND METHODS

### Literature search

Two reviewers (LY and MJ) independently undertook a computer-aided search of the MEDLINE and EMBASE databases to identify relevant studies (last update July 30, 2017), with the following combination of search terms: second primary cancers, synchronous cancers, secondary cancers, PET, positron emission tomography. The reference lists of all included studies were also screened for potentially eligible studies. Authors of eligible studies were also contacted for supplementing additional data if the key information was missing.

### Study selection

PET-CT studies that met the following criteria were included: (a) ^18^FDG PET-CT was used to detect all subtypes of SPCs in cancer patients. Studies about^18^FDG PET-CT for the detection of the specific subtype of second primary cancers were excluded. (b) the studies were based on per-patient statistics; (c) totals of true-positives, false-positives, true-negatives, and false-negatives were provided or could be calculated; (d) histopathologic data and/or results of imaging follow-up served as the gold standard; (e) the selected studies included at least 20 patients. (f) When data were presented in more than one article, the article with the most details was chosen. (g) non-original articles (letters, abstracts, interviews, case report, editorials, and comments) were excluded in this meta-analysis.

### Data extraction

Two reviewers (LY and MJ) independently extracted the data from each study, including the first author, time of publication, patient age (mean or median), number of patients included, study design (prospective or retrospective), technical characteristics and interpretation method of PET-CT, execution of the gold standard, and study results (totals of true positives, false positives, true negatives, and false negatives). Two reviewers (LY and MJ) resolved any difference by consensus. To calculate the sensitivity and specificity, a true positive result was considered when PET-CT suggested a SPC that could be confirmed subsequently, whereas a result was considered false positive when a SPC could not be confirmed. A true negative result was considered when no SPC was detected by other imaging modalities during follow-up period. The result was classified as false negative if a SPC was detected by other diagnostic modalities after a negative result of PET-CT.

### Quality assessment

Two reviewers (LY and MJ) independently evaluated the methodological quality of all six included studies using the updated quality assessment tool “Quality Assessment of Diagnostic Accuracy Studies (QUADAS)-2” [9].

### Statistical analysis

We carried out all statistical analyses by Stata 12.0 (Stata Corporation, College Station, TX). We used the bivariate model to obtain weighted overall estimates of diagnostic performance (i.e., sensitivity, specificity, PLR and NLR) as the main outcome measures, and to construct summary receiver operating characteristic (SROC) curves for ^18^FDG PET-CT [10, 11]. We used the summary estimates of sensitivity and specificity for PET-CT obtained in the meta-analysis to calculate the NPVs for PET-CT when the prevalences of SPCs in cancer patients were assumed to be 5%, 10%, and 15%.
